# Retrograde Aortic Valve Migration During Transcatheter Aortic Valve Replacement for Aortic Regurgitation: A Case Report Highlighting the Importance of Prompt Venoarterial Extracorporeal Membrane Oxygenation (VA-ECMO) Initiation

**DOI:** 10.7759/cureus.62266

**Published:** 2024-06-12

**Authors:** Atsuhiro Kitaura, Hiroatsu Sakamoto, Shota Tsukimoto, Haruyuki Yuasa, Yasufumi Nakajima

**Affiliations:** 1 Anesthesiology, Kindai University Faculty of Medicine, Osaka, JPN; 2 Dental Anesthesiology, Kanagawa Dental University, Yokosuka, JPN

**Keywords:** anesthesia, aortic regurgitation, transcatheter aortic valve migration, veno-arterial ecmo, transcatheter aortic valve replacement

## Abstract

Surgical aortic valve replacement (SAVR) is the recommended curative treatment for pure native aortic regurgitation (AR). However, some patients are not suitable for SAVR due to comorbidities or frailty. Transcatheter aortic valve replacement (TAVR) has been reported to offer a better prognosis than medical therapy in AR patients; thus, the use of TAVR for AR may increase in the future. However, the reduced calcification and annulus ring stiffness associated with TAVR may increase the risk of valve migration. Accumulating data on rescue measures in the event of valve migration is necessary.

An 87-year-old female with a history of hypertension and persistent atrial fibrillation presented to our emergency department with dyspnea. The patient was diagnosed with congestive heart failure class IV, according to the New York Heart Association classification, necessitating urgent admission to our cardiac department. Due to the patient’s high surgical risk (Society of Thoracic Surgeons (STS) score 9.17%, Euro2 score 9.55%, frailty 6), the heart team performed TAVR with a right femoral arterial approach. The patient was sedated, and pacing was initiated at 180 bpm. We placed an Edwards SAPIEN 3 valve (Edwards Lifesciences, Irvine, CA, USA) #23 (-1 mL volume, with attached balloon). During the post-deployment procedure, the aortic valve migrated retrogradely into the left ventricle (LV). Despite the occurrence of severe aortic valve regurgitation, the patient's vital signs remained stable. Five minutes after the migration of the aortic valve, venoarterial extracorporeal membrane oxygenation (VA-ECMO) was initiated. A second TAVR valve implantation was then performed. However, after the second valve implantation and the removal of the pre-shaped guidewire (Safari^2^ pre-shaped guidewire extra small, Boston Scientific, Marlborough, MA, USA), the migrated valve became stuck in the left ventricular outflow tract (LVOT) in a reverse position, resulting in severely limited left ventricular ejection. We increased the support provided by VA-ECMO, and surgical conversion to SAVR was performed without experiencing circulatory collapse. Surgical aortic valve replacement was initiated successfully, and withdrawal of the cardiopulmonary bypass (CPB) was performed without complications. The patient was extubated on the first postoperative day (POD), discharged from the ICU on POD 3, and transferred for rehabilitation on POD 27. In summary, the prompt introduction of VA-ECMO was important for avoiding complications and saving the patient’s life following the retrograde migration of the TAVR valve.

## Introduction

Surgical aortic valve replacement (SAVR) is the recommended curative treatment for pure native aortic regurgitation (AR) [1,2]. However, due to its highly invasive nature, SAVR is not feasible for all patients such as patients with older age, comorbidities, or frailty. On the other hand, the prognosis for AR patients without operation is reported to be poor [3]. Transcatheter aortic valve replacement (TAVR) remains a challenging procedure for patients with AR [4-6]. Nevertheless, TAVR with newer generation devices is useful as an alternative curative procedure for patients deemed ineligible for SAVR [4,7,8]. It is reported that TAVR improved the prognosis of AR compared to medical therapy alone [6,9]. On the other hand, aortic valves in AR patients lack calcification in the annulus and cusps, which can cause problems with the anchoring of the prosthetic valve. The risk of valve migration is reported to be higher than in patients with aortic stenosis [5,7,9]. Retrograde valve migration is a rare complication of TAVR in aortic stenosis patients; thus, data accumulation is necessary to ensure the safe expansion of TAVR to AR. Here, we report a case of retrograde migration during TAVR in a patient with AR. Although the patient's vitals were stable, we immediately initiated veno-arterial extracorporeal membrane oxygenation (VA-ECMO), which averted a subsequent critical situation.

## Case presentation

An 87-year-old female (height: 150 cm, body weight: 45 kg) with a history of hypertension and persistent atrial fibrillation presented to our emergency department with dyspnea. She was diagnosed with congestive heart failure class IV per the New York Heart Association classification and was urgently admitted to our cardiac department. She was taking 25 mg spironolactone, 40 mg furosemide, 0.125 mg digoxin, 20 mg famotidine, and 10 mg enalapril maleate, which were to be continued during the perioperative period.

During the physical examination, an early diastolic decrescendo murmur was heard most prominently at the third intercostal space on the right. Subsequent echocardiography confirmed the presence of severe AR and a left ventricular ejection fraction (LVEF) of 0.3. Coronary CT showed no angiographic lesions. However, due to the patient’s high surgical risk (Society of Thoracic Surgeons (STS) score 9.17%, Euro2 score 9.55%, frailty 6), the heart team decided to continue medical treatment. There was no improvement in the patient’s clinical state. Echocardiography after medical treatment revealed residual moderate AR, dense vena contracta measuring 4.2 mm, a pressure half-time of 396 ms, an effective regurgitant orifice area of 0.25 cm2, and a regurgitant volume of 52 mL. Hyperintensity of aortic valve leaflets and annulus were observed. The aortic valve area was 1.5 cm2, and the left ventricular ejection fraction (LVEF) was 34%. It was determined that intervention in the aortic valve was necessary; therefore, we performed TAVR.

**Figure 1:**
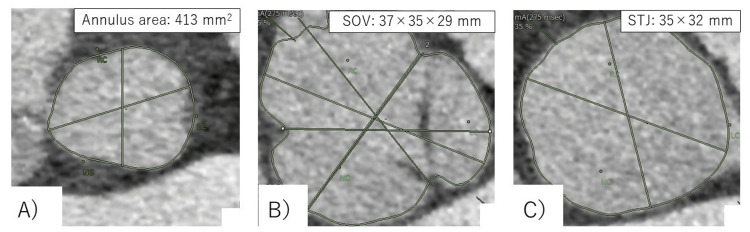
Preoperative CT around the aortic valve A:  Annulus, B: Valsalva, C: Sinotubular junction The CT revealed that the patient's anatomy was compatible with the 23 mm size of the Sapien 3 Ultra RESILIA (Edwards Lifesciences, Irvine, CA, USA). There was virtually no calcification of the aortic annulus or cusp. SOV: Sinus of Valsalva, STJ: Sinotubular junction

During the planning of the procedure, CT revealed a non-coronary sinus diameter of 37 mm, a left coronary sinus diameter of 35 mm, and a right coronary sinus diameter of 413 mm2, with an annulus perimeter of 75.1 mm. Based on the measured values, Evolute PRO+ 29 mm (Medtronic Inc., Minneapolis, MN, USA) was determined to be suitable. The annulus area was calculated to have calcium deposition in the region (Figure 1).

**Figure 2:**
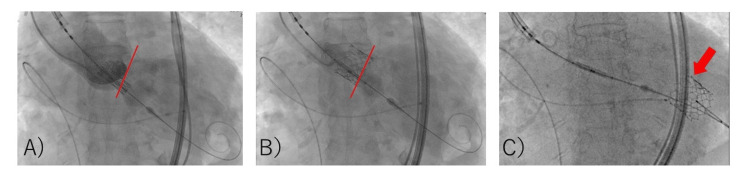
Fluoroscopic images of the first TAVR valve implantation A and B: The TAVR valve (Sapien 3 Ultra RESILIA; Edwards Lifesciences) implantation is completed in a normal position with rapid pacing. C: The first TAVR valve migrates into the left ventricle due to the interference of the reinserted implantation device. The migration valve is captured by a pre-shaped guidewire. Red lines indicate the location of the annulus. The red arrow indicates the migrated valve. TAVR: Transcatheter aortic valve replacement

These characteristics indicated a high risk of migration, so we opted to use the Edwards SAPIEN 3 valve #23. Under sedation with 1 mg/kg/h remimazolam and 0.03 µg/kg/min remifentanil, the Edwards SAPIEN 3 valve #23 was placed using a right femoral arterial approach (pacing at 180 bpm, -1 mL volume, with attached balloon) (Figure 2, A and B).

**Figure 3:**
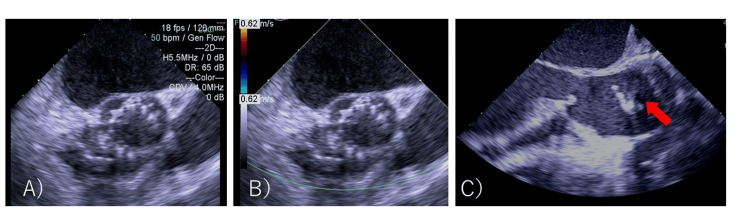
Transesophageal echocardiographic images of the second TAVR valve A: Mid-esophageal short-axis view (B mode) of the TAVR valve (B mode) showing that the second valve is normally implanted B: Short-axis view of the TAVR valve revealed no perivalvular regurgitation detected with color Doppler imaging C: Mid-esophageal aortic valve long-axis view after aortic clamping revealing the anterograde migration of the second valve due to surgical compression. A red arrow indicates the migrated second valve. The first valve is not visible because it has dropped out of the image into the LV due to loss of cardiac output. TAVR: Transcatheter aortic valve replacement, LV: Left ventricle

When the device position was adjusted for post-ballooning, the aortic valve migrated retrogradely into the left ventricle (LV) (Figure 2, C). Attempts to crimp the valve to the left ventricular outflow tract (LVOT) by balloon were unsuccessful. Although severe aortic valve regurgitation occurred, the patient's vitals were stable. The cardiac surgical team was convened, and priming of the heart-lung machine was started immediately. As it was determined that about 40 minutes of preparation were needed, the cardiologist decided to attempt another valve implantation. The anesthesiologist suggested that the VA-ECMO be placed ahead of time. The patient was inserted with an arterial line in the left femoral artery and a venous line in the right femoral vein. Five minutes after migration, VA-ECMO was driven. Anesthesia was converted to general anesthesia. In addition to the remimazolam and remifentanil already administered, 50 mg of rocuronium was administered, and tracheal intubation was performed. General anesthesia was maintained with 1 mg/kg/h remimazolam and 0.03-0.25 µg/kg/min remifentanil. A transesophageal echocardiography (TEE) probe was inserted. Approximately 50 minutes after migration, a second valve replacement (SAPIEN 3 valve #23, nominal volume, with attached balloon) was performed using a right femoral approach. The TEE did not reveal any perivalvular leakage (Figure 3, A and B).

**Figure 4:**
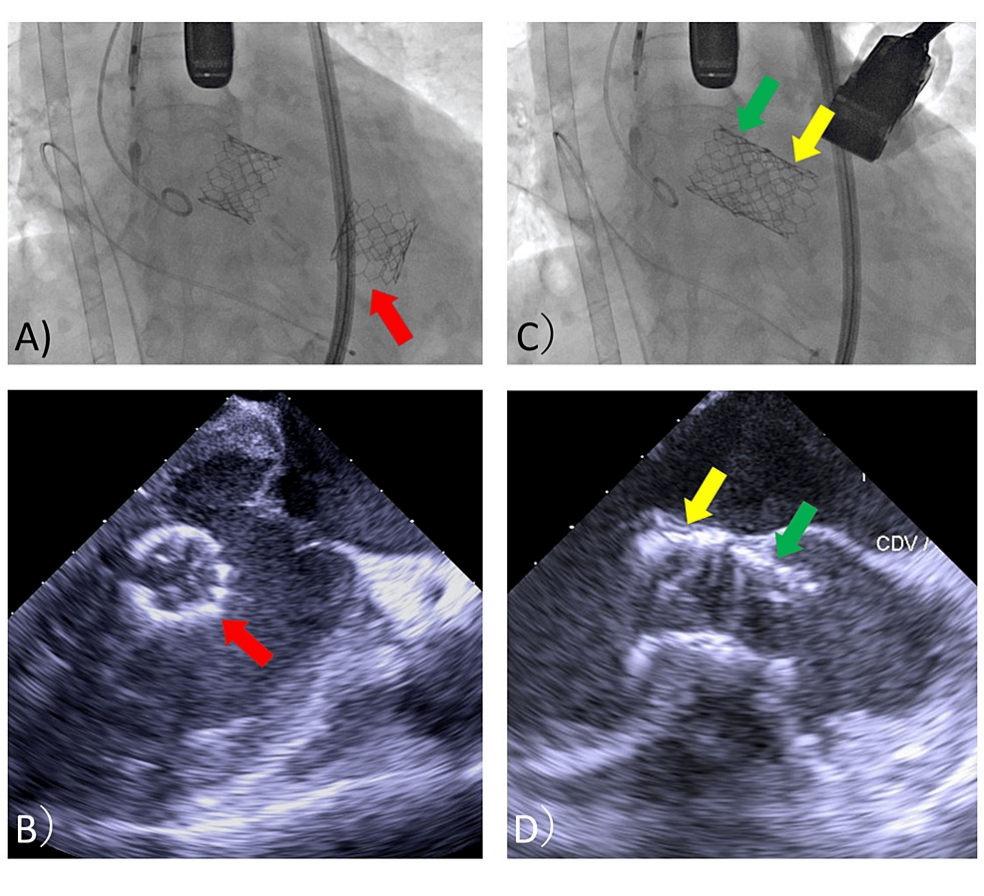
Fluoroscopic and TEE images of the migrated first TAVR valve A and B: A rotating TAVR valve is observed in the LV. Red arrows indicate the retrograde, migrated first valve floating in the LV. C and D: Autonomous insertion of the migrated TAVR valve in the LV outlet in reverse valve position. Yellow arrows indicate the first valve stuck in the LVOT in the reversed position. Green arrows show the second valve implanted in the aortic valve position. TEE: Transesophageal echocardiography, TAVR: Transcatheter aortic valve replacement, LV: Left ventricle, LVOT: Left ventricular outflow tract

Bleeding at the delivery sheath insertion site was observed after the second valve deployment. Fearing bleeding during the surgery with cardiopulmonary bypass (CPB), the sheath and wire were removed, and hemostasis was performed at the puncture site using a hemostatic device. After removal of the pre-shaped guidewire (Safari2 pre-shaped guidewire extra small; Boston Scientific, Marlborough, MA, USA), rotational movement of the first valve was observed in the ventricular cavity (Figure 4, A and B).

**Figure 5:**
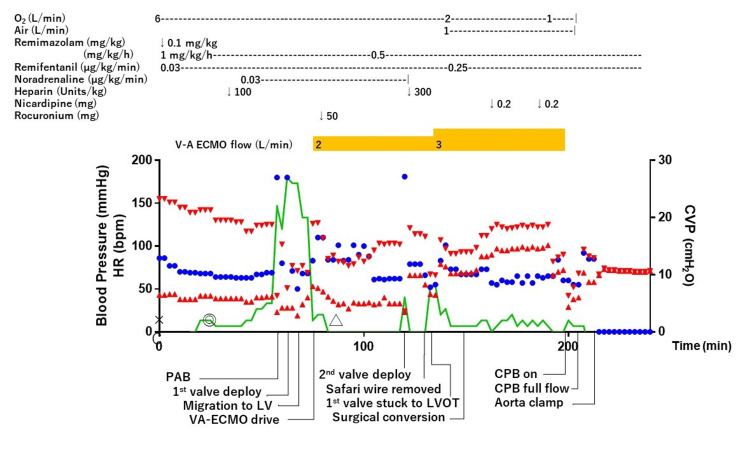
Anesthesia chart The patient’s vitals became unstable due to the migration of the first valve but did not collapse. The recovery procedure was implemented while maintaining stable hemodynamics with VA-ECMO. The first valve was stuck in the LVOT in the opposite direction, making auto cardiac output difficult; however, this was immediately addressed by increasing VA-ECMO flow. Blue dots indicate heart rate. Red inverted triangles indicate systolic blood pressure. Red upright triangles also show diastolic blood pressure. The green line indicates the trend of CVP. VA-ECMO: Venoarterial extracorporeal membrane oxygenation, LVOT: Left ventricular outflow tract, HR: Heart rate, CVP: Central venous pressure, PAB: Pulmonary artery banding, CPB: Cardiopulmonary bypass, LV: Left ventricle

A few minutes after the deployment of the second valve, the first valve was autonomously introduced into the LV outlet in a reversed position (Figure 4, C and D). After this unexpected complication, the patient’s cardiac output was severely impaired. However, because the ECMO was being driven, the patient’s vitals could be managed without fluctuation. Surgical removal of the migration valve in her LV was initiated. A median sternotomy was made, an arterial line was inserted into the ascending aorta, the venous line of VA-ECMO was attached to the CPB circuit, and CPB was established. The anesthesia chart until CPB establishment is shown in Figure 5.

**Figure 6:**
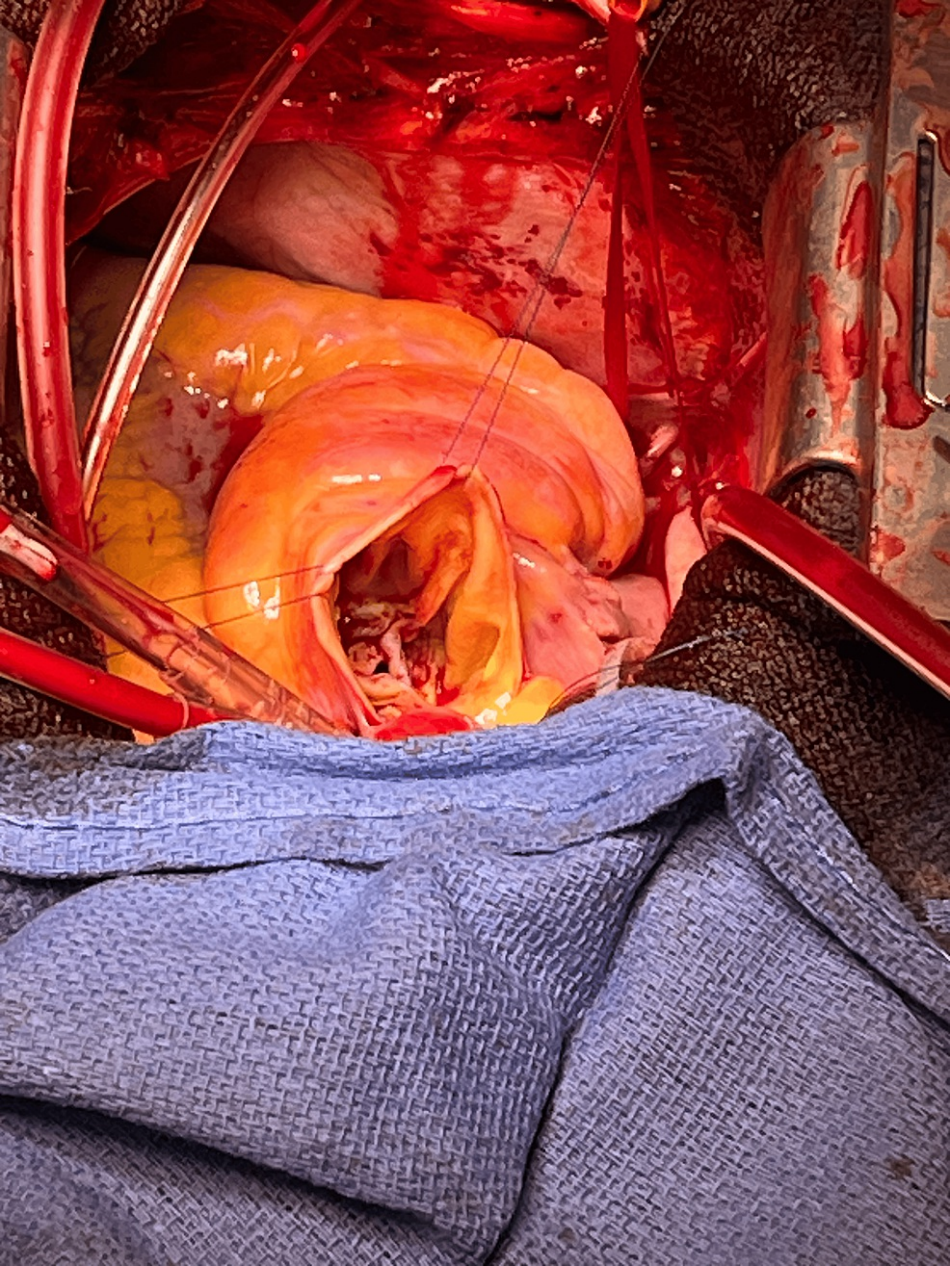
Intraoperative view of the patient’s native aortic valve A native tricuspid aortic valve without calcification was identified. The patient's aortic valve cusps were slightly thickened, but there was no calcification. The annulus ring, too, had no calcification and was flexible.

Immediately after aorta clamping, prograde migration of the second valve occurred (Figure 3, C). Therefore, the original plan to remove the first valve in the LV by transseptal approach was changed to a transaortic approach. After aortomy, the two prolapsed valves were removed from the aortic side. There was no calcification of the aortic valve and valve ring, and the valve ring was flexible (Figure 6).

A bioprosthetic valve (Inspiris 23 mm; Edwards Lifesciences) was implanted. Withdrawal of the CPB was performed without complications. The patient was extubated on the first postoperative day (POD), discharged from the ICU on POD 3, and transferred for rehabilitation on POD 27.

## Discussion

In the present case, SAVR was considered to be high risk due to the patient’s age and frailty; thus, we performed TAVR. Surgical aortic valve replacement is the first choice for surgical treatment of AR [1,2]. However, it is difficult to adapt to all patients because of its invasiveness. Transcatheter aortic valve replacement for AR has been reported to have a better prognosis than medication [6,9]. In patients with high-risk severe aortic atresia, the one-year mortality rate was reported to be 20% with medical therapy, compared to 10% with TAVR [10,11]. In addition, some reports suggest that TAVR may be safer than SAVR [12]. Hence, TAVR for AR may increase in the future. On the other hand, TAVR in patients with AR is associated with an increased risk of migration, possibly due to less calcification of the aortic valve cusps and less sclerosis of the annulus, making it more difficult to anchor the TAVR valve [5,7,9]. Depending on the generation of the device, the rate of valve migration requiring a second valve implantation has been reported as 7% to 12% [6,9]. Therefore, it is anticipated that the number of cases of TAVR valve migration will increase in the future. Further studies are needed to establish procedures to respond to TAVR valve prolapse.

Retraction and re-impaction using an oversized balloon have been reported as a technique to recover from valve migration [13-15]. However, in the present case, the attempt to reposition the first valve at the annulus was unsuccessful. It has also been reported that second valve implantation as a recovery to valve migration is effective in controlling acute AR and reduces the invasiveness of open-heart surgery by avoiding SAVR [16,17]. In the present study, a second valve implantation was performed while preparing for SAVR. However, during the surgical procedure, the implanted second valve dislodged. Although physical pressure from the surgical operation may have contributed to this migration, intraoperative findings indicated minimal calcium in the aortic valve and potential issues with valve anchoring. Consequently, it was concluded that SAVR was the appropriate course of action in this case. In the present case, it was initially determined that the patient should avoid SAVR due to the high risk involved. However, in reality, the patient was able to tolerate the more stressful situation presented in this instance than a typical SAVR procedure. Given that SAVR is the first-line treatment for AR, it is still difficult to accurately determine a priori, but in the present case it may have been necessary to carefully evaluate the likelihood of a patient receiving SAVR.

At the time of the first valve migration, the patient's vital signs remained stable. This was considered fortunate, as the patient exhibited tolerance to AR due to the original presence of severe AR. Additionally, the prolapsed valve did not restrict left ventricular wall motion, likely due to left ventricular enlargement. However, we immediately introduced VA-ECMO. The rationale for using VA-ECMO was based on two factors. First was the predicted instability of vitals with subsequent procedures. The second was because the efficacy and safety of scheduled VA-ECMO in high-risk TAVR had been reported [18]. Although TAVR can be performed more quickly than SAVR with cardiopulmonary priming, it requires time to prepare a new valve for implantation, and as in this case, there was a possibility of embolization due to a prolapsed valve or even a prolapsed second valve. Therefore, it was considered safe to immediately perform assisted circulation to ensure hemodynamic stability. There have been reports that VA-ECMO use may be considered in the event of valve migration [14]. Prophylactic use of VA-ECMO was considered an option that could safely maintain hemodynamic status [19]. Eventually, the migrated prosthesis became stuck in the LVOT about 50 minutes after migration, rendering left ventricular ejection nearly impossible. As a migrating valve can migrate by the bloodstream, it may become an embolus when positioned in a reverse or sideways orientation, potentially resulting in circulatory failure [16]. In the present case, we were able to maintain the patient's vitals by increasing the level of ECMO assistance. In cases of retrograde migration of the prosthesis, the pre-shaped guidewire should be removed after establishing assisted circulatory support, despite the obstacles to aortic clamping. This is because as long as the guidewire captures the prolapsed valve, the prolapsed valve will not remain lodged in the LVOT in the reverse position. Although our case was considered acceptable for early wire removal because the VA-ECMO was already driven and circulation was assured, one must usually be cautious about removing the wire. As a result, the decision to quickly introduce VA-ECMO was considered to have saved the patient from a potentially fatal complication.

## Conclusions

We encountered a case of TAVR valve migration that was salvaged through the prompt introduction of supplemental circulation, which ultimately led to surgical intervention. Based on the favorable results, our case, although a high-risk case, may have required more prudence in the decision to avoid SAVR. The rapid initiation of supplemental circulation was effective in stabilizing the patient's hemodynamics, thereby facilitating the management of potential subsequent complications. Delays in initiating supplemental circulation should be avoided in similar scenarios. Therefore, at our institution, we have changed our practice to prioritize extracorporeal circulatory support first in the event of retrograde migration of the TAVR valve. In particular, it was deemed necessary to ensure extracorporeal circulatory support before removing the guidewire while enabling the capture of the migrated prosthesis.
